# Identification of brain-enriched proteins in the cerebrospinal fluid proteome by LC-MS/MS profiling and mining of the Human Protein Atlas

**DOI:** 10.1186/s12014-016-9111-3

**Published:** 2016-05-15

**Authors:** Ilijana Begcevic, Davor Brinc, Andrei P. Drabovich, Ihor Batruch, Eleftherios P. Diamandis

**Affiliations:** Department of Laboratory Medicine and Pathobiology, University of Toronto, Toronto, ON Canada; Lunenfeld-Tanenbaum Research Institute, Mount Sinai Hospital, Toronto, ON Canada; Department of Clinical Biochemistry, University Health Network, Toronto, ON Canada

**Keywords:** Cerebrospinal fluid, Human Protein Atlas, Mass spectrometry, LC-MS/MS, Brain-enriched proteins

## Abstract

**Background:**

Cerebrospinal fluid (CSF) is a proximal fluid which communicates closely with brain tissue, contains numerous brain-derived proteins and thus represents a promising fluid for discovery of biomarkers of central nervous system (CNS) diseases. The main purpose of this study was to generate an extensive CSF proteome and define brain-related proteins identified in CSF, suitable for development of diagnostic assays.

**Methods:**

Six non-pathological CSF samples from three female and three male individuals were selected for CSF analysis. Samples were first subjected to strong cation exchange chromatography, followed by LC-MS/MS analysis. Secreted and membrane-bound proteins enriched in the brain tissues were retrieved from the Human Protein Atlas.

**Results:**

In total, 2615 proteins were identified in the CSF. The number of proteins identified per individual sample ranged from 1109 to 1421, with inter-individual variability between six samples of 21 %. Based on the Human Protein Atlas, 78 brain-specific proteins found in CSF samples were proposed as a signature of brain-enriched proteins in CSF.

**Conclusion:**

A combination of Human Protein Atlas database and experimental search of proteins in specific body fluid can be applied as an initial step in search for disease biomarkers specific for a particular tissue. This signature may be of significant interest for development of novel diagnostics of CNS diseases and identification of drug targets.

**Electronic supplementary material:**

The online version of this article (doi:10.1186/s12014-016-9111-3) contains supplementary material, which is available to authorized users.

## Background

Cerebrospinal fluid (CSF) is a proximal fluid residing in direct contact with the cerebral parenchyma. CSF acts to protect, support and nurture brain tissues and is essential for brain functioning. Apart from hydro-mechanical protection, CSF is also important for the homeostasis of the extracellular environment and hormonal-to-neuropeptide balance in the central nervous system (CNS) [1, 2]. The majority of CSF is produced as plasma ultra-filtrate by the choroid plexus in the lateral, third and fourth ventricles, whereas a smaller portion is derived from the cerebral interstitial fluid and cerebral capillaries [3]. CSF production is a dynamic process with a rate of about 500 mL per day, and CSF absorption is mainly performed through arachnoid villi from the subarachnoid space into the venous sinuses [3]. Approximately 80 % of the total CSF protein is derived from the plasma, upon crossing the blood–brain barrier, and another 20 % is secreted by the CNS [1]. Examples of proteins with higher CSF concentration and high CSF-to-blood serum ratios include prostaglandin D2 synthase (ratio 34/1), S-100B (18/1), tau protein (10/1), and cystatin C (5/1) [4, 5]. The most abundant blood-derived proteins in CSF are albumin and immunoglobulins. Blood-related proteins in CSF such as apolipoprotein B-100 and hemoglobin are commonly used as an indication of blood contamination of CSF [1, 6].

Detailed composition of the CSF proteome may provide novel insights for the in-depth understanding of CNS functioning under physiological and pathological conditions. The advantages of tissue-specific proteomes have been previously demonstrated for the discovery of novel protein biomarkers [7, 8]. The Human Protein Atlas (HPA) provides comprehensive data on the tissue-specific transcriptome and proteomes, based on the RNA-sequencing analysis of 32 human tissues and immunohistochemistry analysis of 44 tissues, respectively [9]. Apart from the tissue-specific proteomes, HPA includes comprehensive summaries of regulatory, secreted and membrane, cancer-specific and druggable proteomes. This makes HPA an indispensable repository of the human proteome and its applications for disease diagnostics and drug discovery. It is worth noting that brain is the top second organ with the largest number of tissue-specific genes. From the 1134 elevated genes in the brain, 315 are tissue-enriched genes, 226 genes are found to be elevated in a group of 2–7 tissues and 590 genes are annotated as tissue-enhanced genes. Tissue-enriched genes are considered genes with mRNA expression at least five times higher in the cerebral cortex relative to other tissues, while group-enriched genes have mRNA expression at least five times higher in the group of 2–7 tissues, including cerebral cortex, relative to all other tissues. Lastly, tissue-enhanced genes have at least five times higher mRNA expression in brain relative to the average expression in all other tissues. The gene ontology (GO) analysis of the elevated genes indicates that the main functions of brain proteins are synaptic transmission and neurological processes, whereas most of the brain-enriched genes are membrane-bound or secreted proteins. Interestingly, membrane-bound and secreted proteins represent the majority of the CSF proteome and their fraction is much higher in CSF than in blood [1, 10]. Considering that membrane and secreted proteins are overrepresented in the CSF, they could be potentially reliably identified and quantified, which makes them respectable biomarker candidates. Besides, significant amounts of membrane-shed and secreted proteins may be released into proximal fluids (such as CSF); these proteins have been previously suggested as promising biomarker candidates of various diseases [11, 12].

The field of CSF proteomics is constantly expanding and many efforts have been made to characterize the CSF proteome. The most extensive CSF protein mapping to date, by Zhang et al., identified 3256 proteins [10], and by Guldbrandsen et al., identified 3081 proteins [13], followed by Schutzer et al. with 2630 [14], and Pan et al. with 2594 proteins [15].

The main purpose of the present study was to expand the knowledge of the human CSF proteome and generate a panel of brain-enriched proteins that can potentially serve as a platform for biomarker discovery of CNS diseases. Here, we performed two-dimensional chromatography (off-line strong-cation exchange fractionation followed by the on-line reverse-phase separation) and mass spectrometry analysis to generate the extensive proteome of normal CSF samples. HPA data was further applied to select brain-related secreted and membrane-bound proteins found in the CSF. Since high-quality antibodies and ELISAs may not be available for many brain tissue-specific proteins, we provided a list of brain-enriched proteins detectable by mass spectrometry and thus quantifiable in CSF by antibody-free selected reaction monitoring (SRM) assays [16, 17].

## Methods

### Cerebrospinal fluid sample preparation

Six non-pathological (normal) CSF samples were retrospectively retrieved for CSF proteome analysis as samples archived after routine biochemical examinations at the Mount Sinai Hospital, Toronto and stored at −80 °C until further use. All samples were transparent, clear and without any visible blood contamination. The patients’ age ranged from 32 to 72 years and included three female and three male patients. The ethical approval was obtained from the Mount Sinai Hospital Research Ethics Board.

For the CSF proteomic analysis, samples were thawed at room temperature, centrifuged for 10 min at 17,000*g* and subjected to mass spectrometry sample preparation. Each CSF sample was adjusted to a volume equivalent to 300 µg total protein, denatured with 0.05 % RapiGest (Waters, Milford, USA) and reduced with 5 mM dithiothreitol (Sigma-Aldrich, Oakville, Canada) at 60 °C for 40 min. Alkylation was achieved with 15 mM iodoacetamide (Sigma-Aldrich, Oakville, Canada) for 60 min in the dark at room temperature. Protein digestion was carried out with trypsin (Sigma-Aldrich, Oakville, Canada) in 50 mM ammonium bicarbonate (1:30 trypsin to total protein ratio), for 18 h at 37 °C. Digestion and RapiGest cleavage were completed with 1 % trifluoroacetic acid following sample centrifugation at 500*g* for 30 min. Samples were frozen at −80 °C until strong-cation exchange (SCX) HPLC peptide separation.

### Strong cation exchange chromatography

Trypsinized samples were diluted two-fold with the SCX Buffer A (0.26 M formic acid, 5 % acetonotrile) and loaded on the SCX PolySULFOETHYL Column (The Nest Group, Inc, Southborough, USA) coupled to the Agilent 1100 HPLC system. The peptides were eluted with the gradual increase of the SCX Buffer B (0.26 M formic acid, 5 % acetonitrile, 1 M ammonium formate) during the 70 min gradient (30–40 min 20 % SCX Buffer B; 45–55 min 100 % SCX Buffer B) and a flow rate of 200 µL/min. The eluent was monitored at 280 nm and fractions (400 µL) were collected. Based on the elution profile, 15 individual fractions and one pooled fraction (for low absorbance fractions, at the end of the gradient) per sample were selected for mass spectrometry analysis. Peptides were purified by extraction using OMIX C18 tips, eluted with 5 µL of acetonitrile solution (65 % acetonitrile, 0.1 % formic acid) and finally diluted with 60 µL of water-formic acid (0.01 % formic acid) solution.

### Liquid chromatography–tandem mass spectrometry (LC-MS/MS)

In total, 96 desalted SCX fractions from six individual CSF samples were loaded on the 96 well-plate. Using an auto-sampler, 18 µL of each sample were injected into an in-house packed 3.3 cm trap pre-column (5 μm C18 particle, column inner diameter 150 μm) and peptides were eluted from the 15 cm analytical column (3 μm C18 particle, inner diameter 75 μm, tip diameter 8 μm). The liquid chromatography, EASY-nLC system (Thermo Fisher, Odense, Denmark) was coupled online to the Q-Exactive Plus (Thermo Fischer, San Jose, USA) mass spectrometer with a nanoelectrospray ionization source. The 60-min liquid-chromatography (LC) gradient was applied with an increasing percentage of buffer B (0.1 % formic acid in acetonitrile) for peptide elution; at the flow rate of 300 nL/min. Full MS1 scan was acquired from 400 to 1500 m/z in the Orbitrap at a resolution of 70,000, followed by the MS2 scans on the top 12 precursor ions at a resolution of 17,500 in a data-dependent acquisition (DDA) mode. The dynamic exclusion was enabled for 45 s and unassigned charge, as well as charge states +1 and +4 to ≥8 were omitted from MS2 fragmentation.

### Data analysis

The Human Protein Atlas (HPA) [9] version 13 (the tissue specific proteome database) was utilized to generate a list of secreted and membrane-bound brain-expressed proteins that had high mRNA expression in the brain relative to other human tissues. The list of 318 brain-enriched proteins (with mRNA expression at least 5 times higher in the cerebral cortex relative to other tissues) and 226 group-enriched proteins (with mRNA expression at least 5 times higher in the group of 2–7 tissues, including cerebral cortex) was downloaded from the HPA database (www.proteinatlas.org). Brain-related proteins were then merged with the secretome (n = 3171 proteins) and the membrane proteome (n = 5570 proteins), generated based on the prediction algorithms for membrane and secreted proteins. Immunohistochemistry-based expression (IHC) of the candidate proteins were manually assessed using the HPA database. Validation data were annotated as IHC brain evidence (detected, not detected, NA—not available) and IHC tissue expression (number of tissues protein is expressed/total number of tissues evaluated), considering four brain-derived tissues as a single tissue.

Raw files were uploaded into the Proteome Discoverer, version 1.4 (Thermo Fischer, San Jose, USA), and searched with both Mascot and Sequest HT algorithms against the human TrEMBL database (July 2014 release). Searching parameters included: two maximum missed cleavages, cysteine carbamidomethylation as a static modification, methionine oxidation as a dynamic modification, precursor mass tolerance of 7 ppm, fragment mass tolerance of 0.02 Da. Proteins were grouped automatically by Proteome Discoverer software and the master protein per group was assigned by the Parsimony Principle. Decoy database search was set to 1 % false discovery rate at the peptide level. The final list of brain-enriched and group-enriched candidates was selected based on protein identification in at least 4 out of 6 individual samples. Brain-enriched (n = 196) and group-enriched (n = 138) proteins were first retrieved from HPA, merged with secreted/membrane proteome to generate a list of brain/group enriched secreted/membrane proteins which were then merged with the in-house generated CSF proteome (based on the gene name) using R statistical software version 2.15.2 (www.Rproject.org). Label-free quantification of the CSF proteome and 78 candidate proteins was performed using MS1 area obtained with Proteome Discoverer (v1.4). Venn diagram for inter-individual sample reproducibility was prepared using Jvenn [18]. The GO analysis of candidate proteins was executed with PANTHER classification system [19]. The comparison between in-house developed CSF proteome and CSF proteome from the literature (Guldbrandsen et al. and Zhang et al.) was performed with R statistical software (v 2.15.2), merging UniProt accession protein identifiers.

## Results

### Cerebrospinal fluid proteome

**Fig. 1:**
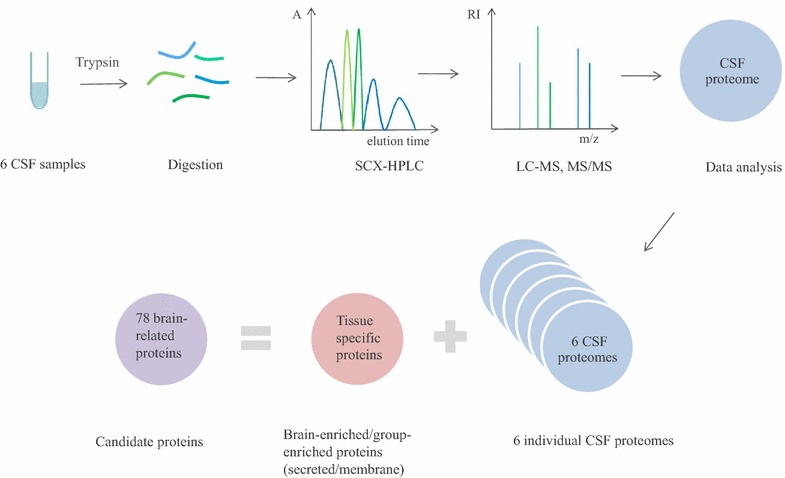
Candidate selection workflow. Six individual CSF samples were digested, fractionated with SCX–HPLC and analyzed with LC-MS, MS/MS to generate in-house human CSF proteome. 196 brain-enriched and 138 group-enriched proteins (secreted/membrane) were compared against individual CSF proteome and 78 brain-related proteins, found reproducibly in individual CSF samples, were selected. *A* absorbance, *RI* relative intensity, *m/z* mass-to-charge ratio

To generate an in-house CSF proteome of wide age range of healthy individuals, six non-pathological CSF samples from three female and three male individuals were selected (Fig. 1), with patients’ age from 32 to 72 years. Numbers of identified proteins in each individual CSF sample ranged from 1109 to 1421, while numbers of identified peptides varied from 6272 to 8632 at 1 % FDR at the peptide level. Merging of proteomes of six individuals resulted in 2615 proteins (12,443 peptides) which represented our complete CSF proteome. Table 1 includes the number of proteins and peptides identified in all 6 CSF samples.

**Table 1:** Number of identified proteins and peptides in six individual CSF samples

Sample	# Proteins	# Peptides
CSF1	1200	6978
CSF2	1282	7629
CSF3	1109	6272
CSF4	1421	8632
CSF5	1305	7253
CSF6	1241	6756
Total	2615	12,443

**Fig. 2:**
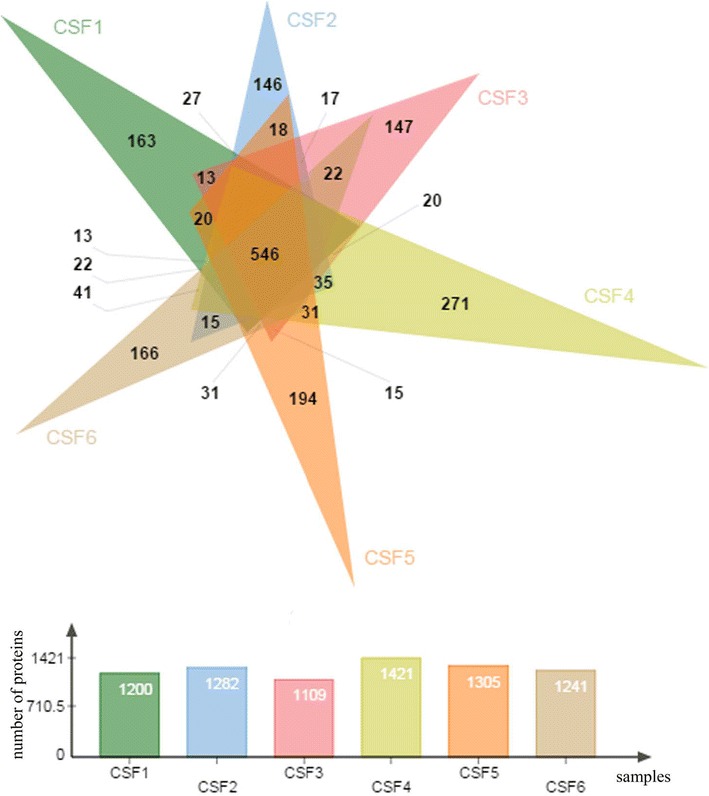
Venn diagram of proteins identified in 6 individual CSF samples. Total number of identified proteins in all samples was 2615 with 546 (21 %) common proteins for all 6 samples. Number of individual proteins ranged from 1109 to 1421

**Fig. 3:**
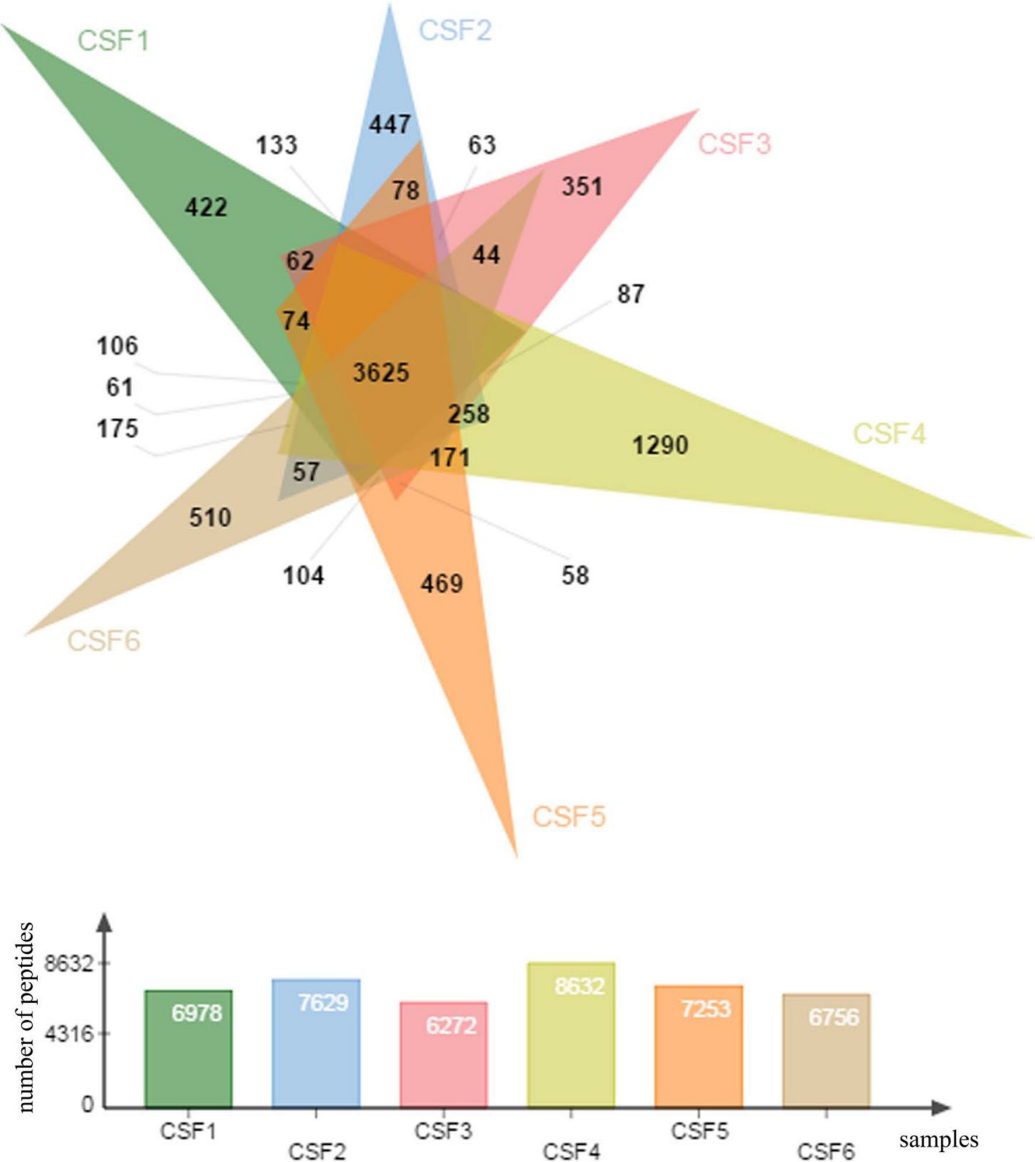
Venn diagram of peptides identified in 6 individual CSF samples. Total number of identified proteins in all samples was 12,443 with 3625 (29 %) common proteins for all 6 samples. Number of individual proteins ranged from 6272 to 8632

Between any two samples, the average percentage of common proteins was 66.9 %. Fewer proteins were common between 3 and 6 samples. Specifically, 1183 (45.4 %) proteins were common in at least 3 samples, 947 (36.2 %) in at least 4 samples, 734 (28.1 %) proteins were shared with at least 5 samples, while 546 (20.9 %) were shared among all 6 samples (Fig. 2; Table 2; Additional file 1: figure 1). At the peptide level, the average percentage of peptides common between any two samples was 74 %. Similar to proteins, fewer number of peptides where common among more samples. 7423 (59.7 %) identified peptides were shared among at least 3 samples, 6138 (49.3 %) between at least 4 samples, 4937 (39.7 %) peptides between at least 5 samples, while 3625 (29.1 %) were shared among all 6 samples (Fig. 3; Table 3; Additional file 2: figure 2).

**Table 2:** Overlap of proteins in individual samples

Overlapped CSF A and B	Number	Percentage of A	Percentage of B
CSF1 and CSF2	859	71.6	67.0
CSF1 and CSF3	757	63.1	68.3
CSF1 and CSF4	823	68.6	57.9
CSF1 and CSF5	827	68.9	63.4
CSF1 and CSF6	787	65.6	63.4
CSF2 and CSF3	794	61.9	71.6
CSF2 and CSF4	924	72.1	65.0
CSF2 and CSF5	900	70.2	69.0
CSF2 and CSF6	841	65.6	67.8
CSF3 and CSF4	780	70.3	54.9
CSF3 and CSF4	771	69.5	59.1
CSF3 and CSF6	752	67.8	60.6
CSF4 and CSF5	894	62.9	68.5
CSF4 and CSF6	859	60.5	69.2
CSF5 and CSF6	843	64.6	67.9
Common in all 6 CSFs^a^	546	20.9	NA
Common in at least 5 CSFs	734	28.1	NA
Common in at least 4 CSFs	947	36.2	NA
Common in at least 3 CSFs	1188	45.4	NA

^a^Among 2615 proteins

**Table 3:** Overlap of peptides in individual samples

Overlapped CSF A and B	Number	Percentage of A	Percentage of B
CSF1 and CSF2	5696	81.6	74.7
CSF1 and CSF3	4982	71.4	79.4
CSF1 and CSF4	5502	78.8	63.7
CSF1 and CSF5	5367	76.9	74.0
CSF1 and CSF6	5003	71.7	74.1
CSF2 and CSF3	5180	67.9	82.6
CSF2 and CSF4	6181	81.0	71.6
CSF2 and CSF5	5748	75.3	79.2
CSF2 and CSF6	5276	69.2	78.1
CSF3 and CSF4	5102	81.3	59.1
CSF3 and CSF5	4988	79.5	68.8
CSF3 and CSF6	4633	73.9	68.6
CSF4 and CSF5	5815	67.4	80.2
CSF4 and CSF6	5415	62.7	80.2
CSF5 and CSF6	5199	71.7	77.0
Common in all 6 CSFs^a^	3625	29.1	NA
Common in at least 5 CSFs	4937	39.7	NA
Common in at least 4 CSFs	6138	49.3	NA
Common in at least 3 CSFs	7423	59.7	NA

^a^Among 2615 proteins

### Identification of brain-related proteins in the CSF proteome

According to our analysis, the total number of tissue-enriched and group-enriched proteins with HPA evidence of high mRNA expression in the brain was 318 and 226, respectively. Of those, 196 tissue-enriched and 138 group-enriched proteins were secreted and/or membrane proteins (Additional file 3: Table 1, Additional file 4: Table 2).We then examined our CSF proteome for the presence of those 196 tissue-enriched and 138 group-enriched proteins (Fig. 1).

**Fig. 4:**
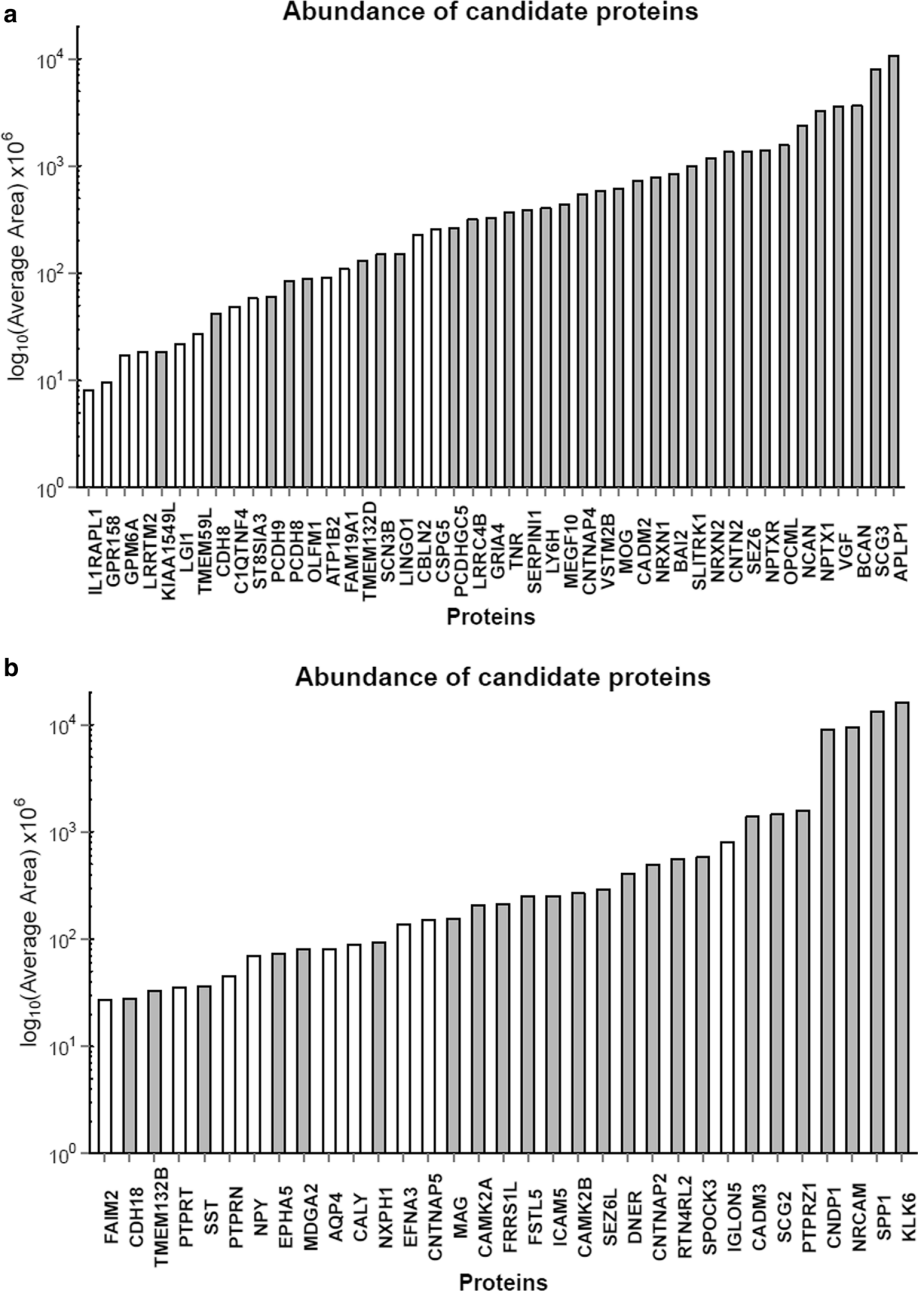
CSF brain tissue-enriched and group-enriched proteins and their relative abundance. **a** 45 brain-enriched and **b** 33 group-enriched proteins were detected in at least 4 out of 6 CSFs samples, and the average MS1 area was used as a proxy of protein abundance. Abundance is indicated for representative protein isoform. *Shaded bars* show proteins that are detected in all 6 samples

**Fig. 5:**
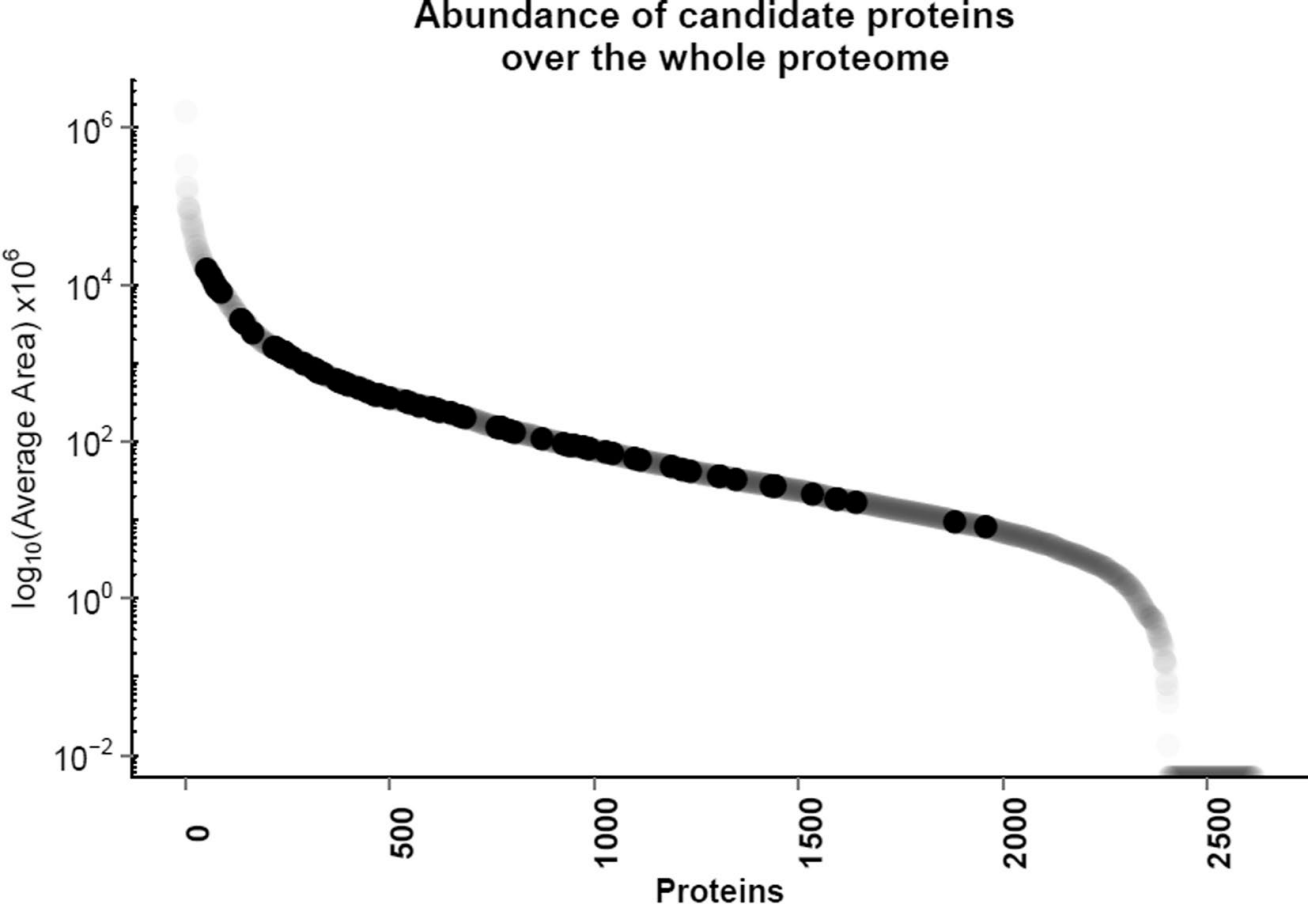
Relative abundance of CSF proteome and 78 protein candidates. *Shaded dots* show 78 protein candidates over the complete proteome. Selected 78 brain-specific proteins were positioned in the range of medium- and upper-abundance proteins of the CSF proteome

**Fig. 6:**
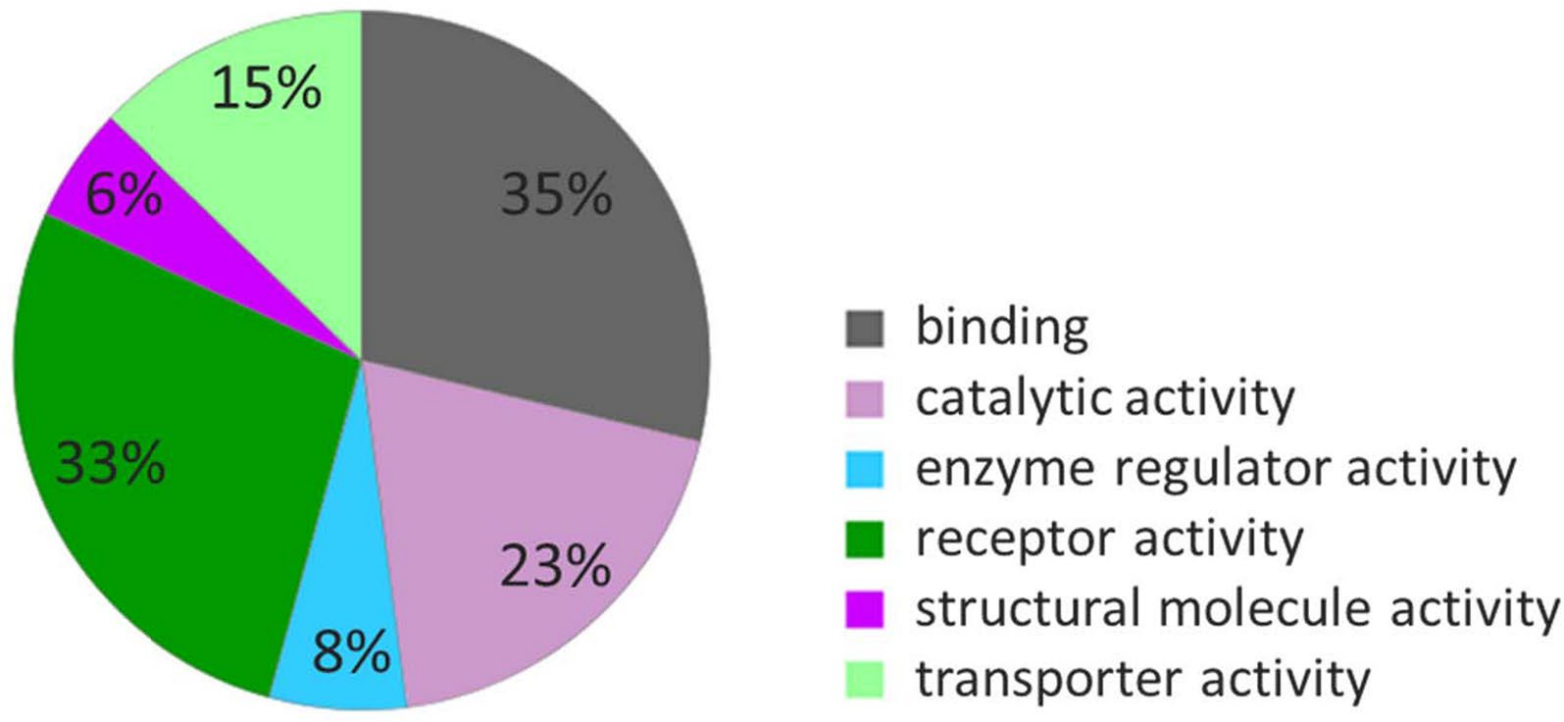
GO analysis of 78 protein candidates. The most represented GO molecular functions were binding (35 % of proteins) and receptor activity (33 % of proteins)

Less than 30 % of brain-enriched (33 proteins) or group-specific proteins (24) were found in all six CSF replicates. Additional proteins can be found in at least 4 or 5 out of the 6 replicate samples. In total, 78 brain-related proteins (secreted or membrane-bound) were found in CSF of at least 4 different individuals. Additional file 5: Table 3 contains the list of all proteins with their relative abundance in CSF based on average area (AA), average number of unique peptides, RNA tissue-specific score (RNA TS) and IHC evidence based on HPA. Based on these experimental data, tissue-enriched proteins with the highest abundance in CSF were amyloid-like protein 1, APLP1 (AA = 1.07 × 10^10^) with average number of 9 unique peptides identified, followed by secretogranin-3, SCG3 (AA = 7.98 × 10^9^ and 24 unique peptides). Of the HPA proteins identified in CSF, V-set and transmembrane domain-containing protein 2B, VSTM2B (RNA TS = 108) and neurocan core protein, NCAN (RNA TS = 60) had the highest RNA TS. In the group-enriched proteins, the most abundant proteins were kallikrein-6, KLK6 (AA = 1.61 × 10^10^; 14 unique peptides) and secreted phosphoprotein 1/osteopontin, SPP1 (AA = 1.32 × 10^10^; 10 unique peptides). Neurexophilin-1, NXPH1 (RNA TS = 44) and contactin-associated protein-like 5, CNTNAP5 (RNA TS = 16) had the highest RNA TS. Figure 4 shows tissue-enriched and group-enriched candidates and their abundance in CSF. In addition, the validation of the KLK6 at the protein level in brain tissues and CSF pool was performed using SRM assay. These findings, together with the methods used, were reported in the Additional file 6: Supplementary method and Additional file 7: figure 3. To compare the relative abundance (based on MS1 area) of selected 78 proteins over the relative abundance of the complete CSF proteome, we plotted MS1 areas of candidate proteins over the MS1 area of all identified proteins (Fig. 5). As a result, most of 78 proteins were positioned in the middle and the upper range of the complete CSF proteome relative abundance. The indication of such candidate distribution suggests that the abundance of the 78 proteins is medium to high when compared to the CSF proteome and thus will be measurable by SRM assays in CSF samples. Knowledge of protein abundances is important to predict if proteins could be quantified in clinical samples using SRM assays, as we previously demonstrated for testis-specific proteins in seminal plasma [16]. The most represented GO molecular functions of 78 proteins were binding (35 % of proteins) and receptor activity (33 % of proteins) as shown in Fig. 6.

### Cell type-specific brain-related proteins in the CSF proteome

Given that HPA also contains data on IHC staining of proteins in several brain regions (hippocampus, lateral ventricle, cortex and cerebellum) and cell types, we analyzed the CSF proteins in order to identify brain region- and cell-type specific proteins. Since some CNS diseases originate in specific regions [20, 21] or cell types [21], measurement of CSF proteins with specific expression in the corresponding regions or cell types may pinpoint the pathological process with high diagnostic sensitivity. Proteins with staining specific for a single cell type are shown in Table 4. The neuron-specific proteins included neurosecretory protein VGF, receptor-type tyrosine-protein phosphatase-like N and neurexophilin-1, neuropil specific, neurocan core protein, tenascin-R and cell adhesion molecule 3, while protein with specific staining for the Purkinje cells was transmembrane protein 132D. Immunohistochemical images of these proteins can be found at the HPA website (http://www.proteinatlas.org).

**Table 4:** Representative cell type-specific brain-expressed proteins according to the Human Protein Atlas immunohistochemistry data

Cell type	Gene name	Staining level	Brain region
Neuron	VGF	Medium	Ctx, Hp, LV
	PTPRN	Medium	Hp
		Low	Ctx
	NXPH1	Low	Hp
Neuropil	NCAN	Medium	Ctx
	TNR	Medium	Ctx
	CADM3	Medium	Ctx
Purkinje cell	TMEM132D	Low	Cb

*Ctx* cerebral cortex, *Hp* hippocampus, *LV* lateral ventricles, *Cb* cerebellum

## Discussion

The prime goal of this study was to generate comprehensive proteome of normal CSF samples and define brain-related proteins identified in the generated proteome. In order to obtain in-depth proteome coverage of normal CSF and allow for identification of low abundance proteins, we performed off-line SCX fractionation of individual CSF samples, followed by LC-MS/MS analysis. The Q Exactive Plus mass spectrometer provided high-resolution, high mass accuracy, wide dynamic range and excellent sensitivity, and along with the benefit of pre-fractionation strategy, facilitated identification of the extensive CSF proteome. With a total number of 2615 identified proteins, this study provides additional information about the CSF proteome when compared to previous proteomic studies [13–15, 22–24]. Recent studies of CSF identified similar number of proteins, utilizing different separation methodologies and mass spectrometry-based proteomics [10, 13–15].

We compared our CSF proteome to the CSF proteome identified by Guldbrandsen et al., with 3081 protein sets or 2875 protein groups reported (available from: http://probe.uib.no/csf-pr) and by Zhang et al. with 2513 proteins reported with at least two unique peptides. When CSF proteins from both studies were compared against our proteome, the combined CSF proteome consisted of 4649 proteins and 4346 proteins for Guldbrandsen et al. plus our proteome and Zhang et al. plus our proteome, respectively. Overall, the combined CSF proteome for all three studies consisted of 5133 proteins. The number of overlapping proteins between Guldbrandsen and our study was 819 (18 % of the combined proteomes, 31 % of our proteome), with 2034 proteins detected only in the Guldbrandsen study, and 1796 only in the present study. Similarly, the number of overlapping proteins between Zhang et al. and our study was 782 (18 % of the combined proteomes, 30 % of our proteome), with 1731 proteins detected only in Zhang study, and 1833 only in the present study. In addition, number of unique proteins, identified only in this study, was 1764. These discrepancies in CSF proteins are partially due to the different proteomic workflows and other technical differences. For example, Guldbrandsen et al. used three different separation approaches (immuno-depletion, SDS-PAGE, MM PR-AX, glycoprotein enrichment) while we used a single (SCX) strategy. However, inter-individual variation of CSF composition seems to be the major factor since only 21 % of our identified proteins were common in all 6 samples. The fact that there are numerous unique proteins identified among the different groups indicates a need for more studies, in order to have a complete picture of the CSF proteome. In addition, pre-analytical variables should be standardized allowing for reliable and comparable proteomic research.

It should also be noted that availability of high-quality clinical samples represents a recognized issue in the field of biomarker discovery [25]. Most of the previous studies employed pools of CSF samples for protein identification. Here, we analyzed individual samples in order to obtain complete CSF proteome and to evaluate inter-individual reproducibility. The biological reproducibility among six individual samples indicated that only 21 % of the proteins were common to all samples, which led us to the conclusion that the inter-individual heterogeneity was an important contributor to variation of CSF proteins, as also observed in previous studies [14, 26]. Some of the inter-individual differences could be explained by sex and age differences [22, 27]. Sample size for this comparison is relatively small and sex differences should be further examined. Although the samples in this study cover a wide age range, the age influence on CSF proteome composition was not within the scope of this study.

CSF analysis can be affected by several pre-analytical parameters, such as variability of sample collection tubes, stability, sample storage and other parameters which should be standardized [28, 29]. One of the common pre-analytical parameters that can affect the CSF protein composition is blood contamination, possibly introduced during the lumbar puncture procedure. Protein concentration in CSF is much lower compared to blood (approximately 150 times lower). Therefore, even a small blood contamination can significantly increase the protein amount in the CSF and have an impact on qualitative and quantitative analysis of CSF proteome. In order to ensure the quality of the CSF samples in this study, visual and biochemical analysis was made and samples with no visible blood contamination or xanthochromia were selected. We also sought to determine the contribution of plasma proteins in our CSF proteome. The database of 1050 plasma proteins generated by Guldbrandsen et al. (http://probe.uib.no/csf-pr) was utilized. The number of proteins common to CSF and blood plasma was 415, indicating that 2200 proteins were unique to the CSF.

CSF communicates closely with brain tissue, and as such it can be considered an ideal specimen for biomarker discovery of CNS diseases and basic neuroscience research. Thus, the following goal of the study was to create a signature of highly specific brain-derived proteins identified in our CSF proteome. HPA-based brain-specific proteome (defined here as combining tissue-enriched and group-enriched proteins from HPA) was utilized for candidate selection. Only proteins of secreted or membrane origin were considered. A list of 78 brain-specific proteins found in at least 4 out of 6 of our CSF proteomes, was generated (Fig. 4; Additional file 5: Table 3). Overall, 57 (52 %) of the brain-related proteins (identified in the CSF proteome) were present in all six individual proteomes, 67 (61 %) in at least 5 proteomes, 78 (72 %) in at least 4 proteomes, 85 (78 %) in at least 3 proteomes and 95 (87 %) in at least 2 proteomes (data not shown). In addition, 95 and 96 % of the candidates were found in the proteome of Zhang et al. and Guldbrandsen et al., respectively. We intend to develop highly accurate and specific SRM assays for their quantification in different neurological diseases, to determine their potential as diagnostic or prognostic biomarkers. For some of the candidates, no commercial antibodies have been developed, resulting in limited information about their distribution and concentration in the brain tissues or CSF (for example, VSTM2B protein previously linked to pathogenesis of ataxia telangiectasia [30]). Furthermore, highly specific brain proteins identified in this study could reveal new pathways or disease mechanisms and lead to discovery of novel therapeutic targets. However, some possible limitations of the biomarker discovery approach utilized in this study should be considered. In a disease state, due to the neuronal cell’s degeneration, some of the intracellular proteins could be released in the extracellular space or secreted into the CSF. Any immune cells recruited to the lesion may also secrete proteins into the CSF, although these would not be considered brain-specific. These proteins would thus remain undetected by our study.

Notably, some of the proteins found in CSF have been previously linked to neurodegenerative diseases. For example, APLP1, is a membrane-bound glycoprotein associated with the synaptic function and a member of a highly conserved gene family, together with amyloid precursor protein (APP) and amyloid precursor-like protein 2 (APP2). Several studies have shown co-localization of APLP1 with APP in control subjects and Alzheimer’s disease brain plaques [31, 32]. In addition, APLP1 is one of the substrates of BACE1, an enzyme involved in Alzheimer’s disease pathology [33]. Finally, a recent study also suggests that APLP1 has significance as a potential biomarker of Parkinson’ disease progression [34]. SCG3, part of the granin family involved in the secretory granule biogenesis and neurotransmitter storage and transport, can be accumulated in the senile plaques of Alzheimer’s disease patients [35]. It has also been reported in the context of Parkinson’s disease, in an in vitro model, where SH-SY5Y cell exposure to the neurotoxin paraquat resulted in decreased SCG3 expression levels [36]. SCG3 and SCG2 were previously evaluated as potential biomarkers of multiple sclerosis and decreased levels were observed for SCG3 and SCG2 in serum and CSF samples of multiple sclerosis patients [37, 38].

Kallikrein 6 (KLK6) was the most abundant protein of the group-enriched proteins. KLK6 is one of the 15-member family of the secreted serine proteases with trypsin-like activity. Among all tissues in the body, KLK6 has the highest expression in the central nervous system and high amounts of KLK6 are present in the CSF [39–41]. It has been suggested that KLK6 may process APP and this way contributes to Alzheimer’s disease pathology [42, 43]. Several other studies found decreased levels of KLK6 in Alzheimer’s disease brain regions (e.g. parietal and frontal cortex) [44, 45]. These findings were previously confirmed by our group at the protein level, indicating lower KLK6 levels in Alzheimer’s disease brain tissue extracts [41, 46]. Studies of KLK6 levels in the CSF are still limited and conflicting, showing both low and high levels of KLK6 in the Alzheimer’s disease CSF samples [41, 47]. Recent findings revealed that α-synuclein, a protein involved in the pathology of Parkinson’s disease, is also a potential KLK6 substrate [48–50]. Even more intriguing is the finding that overexpression of KLK6 in α-synuclein transgenic mouse model leads to clearance of α-synuclein, suggesting a potential therapeutic application [51]. In contrast, elevated levels of KLK6 have been observed in multiple sclerosis patients and its role in the disease pathology has been related to an immuno-inflammatory pathway, particularly by activating PAR receptors, key triggers of inflammatory processes [52–54]. Here, we evaluated KLK6 protein level in the brain tissue extracts and pool of CSF samples as a complement of mRNA expression data from HPA (KLK6 immunohistochemistry from the HPA not available). These findings confirmed its abundance in the CSF, as well as in the brain tissue extracts, where significantly differential levels were observed between brain regions (Additional file 7: figure 3).

Another group-enriched protein connected with neurodegenerative diseases and observed with high abundance is a glycosylated phosphoprotein SPP1. A recent study revealed its potential as a diagnostic biomarker of Parkinson’s disease [34]. SPP1 protein expression was found in neurons, Lewy bodies and microglia of substantia nigra region in Parkinson’s disease, pyramidal neurons of hippocampus in Alzheimer’s disease (with increased levels relative to age-matched controls) and astrocytes within plaques and white matter of multiple sclerosis patients (with increased levels relative to controls) [55–57]. SPP1 levels in CSF were also elevated in Alzheimer’s disease and mild cognitive impairment [58], and multiple sclerosis patients [55, 59].

In conclusion, the present study contributes to the existing knowledge of the human CSF proteome and, in addition, provides a panel of highly specific brain-derived proteins that can be robustly measured in CSF by mass spectrometry assays. In future, we intend to develop quantitative SRM assays for selected 78 proteins and use them as a signature biomarker panel for evaluation of various neurodegenerative diseases.

## Additional files

10.1186/s12014-016-9111-3 Proteins common between two samples. Venn diagrams show common proteins between any two individual CSF samples. The average percentage of common proteins was 66.9 %.
10.1186/s12014-016-9111-3 Peptides common between two samples. Venn diagrams show common proteins between any two individual CSF samples. The average percentage of common peptides was 74 %.
10.1186/s12014-016-9111-3 Brain-enriched proteins of secreted/membrane origin.
10.1186/s12014-016-9111-3 Group-enriched proteins of secreted/membrane origin.
10.1186/s12014-016-9111-3 78 tissue-enriched and group-specific candidates.
10.1186/s12014-016-9111-3 KLK6 selected reaction monitoring (SRM) assay.
10.1186/s12014-016-9111-3 KLK6 concentration in brain tissue extracts and CSF pool. Brain tissue extracts and CSF pool were subjected to mass spectrometry sample preparation and analyzed using TSQ Vantage (brain tissues) and TSQ Quantiva (CSF) mass spectrometers. One-way ANOVA and Bonferroni’s Multiple Comparison Test was performed with GradPad Prism between brain regions, n = 3, *p < 0.05. Data are shown as mean ± standard error of the mean (SEM). TP- total protein, SNc- substantia nigra.

## Abbreviations

CSF: cerebrospinal fluid
CNS: central nervous system
HPA: Human Protein Atlas
GO: gene ontology
SCX: strong-cation exchange
AA: average area
RNA TS: RNA tissue specific score
APLP1: amyloid-like protein 1
SCG3: secretogranin-3
VSTM2B: V-set and transmembrane domain-containing protein 2B
KLK6: kallikrein-6
SPP1: osteopontin
