# Supplementary material for: Identification of brain-enriched proteins in the cerebrospinal fluid proteome by LC-MS/MS profiling and mining of the Human Protein Atlas
Source: Clin Proteomics. 2016 May 15;13:11. doi: 10.1186/s12014-016-9111-3 (PMC4868024; doi:10.1186/s12014-016-9111-3)
Supplement: Supplementary file 2 — 10.1186/s12014-016-9111-3 Peptides common between two samples. Venn diagrams show common proteins between any two individual CSF samples. The average percentage of common peptides was 74 %. [file 12014_2016_9111_MOESM2_ESM.pdf]

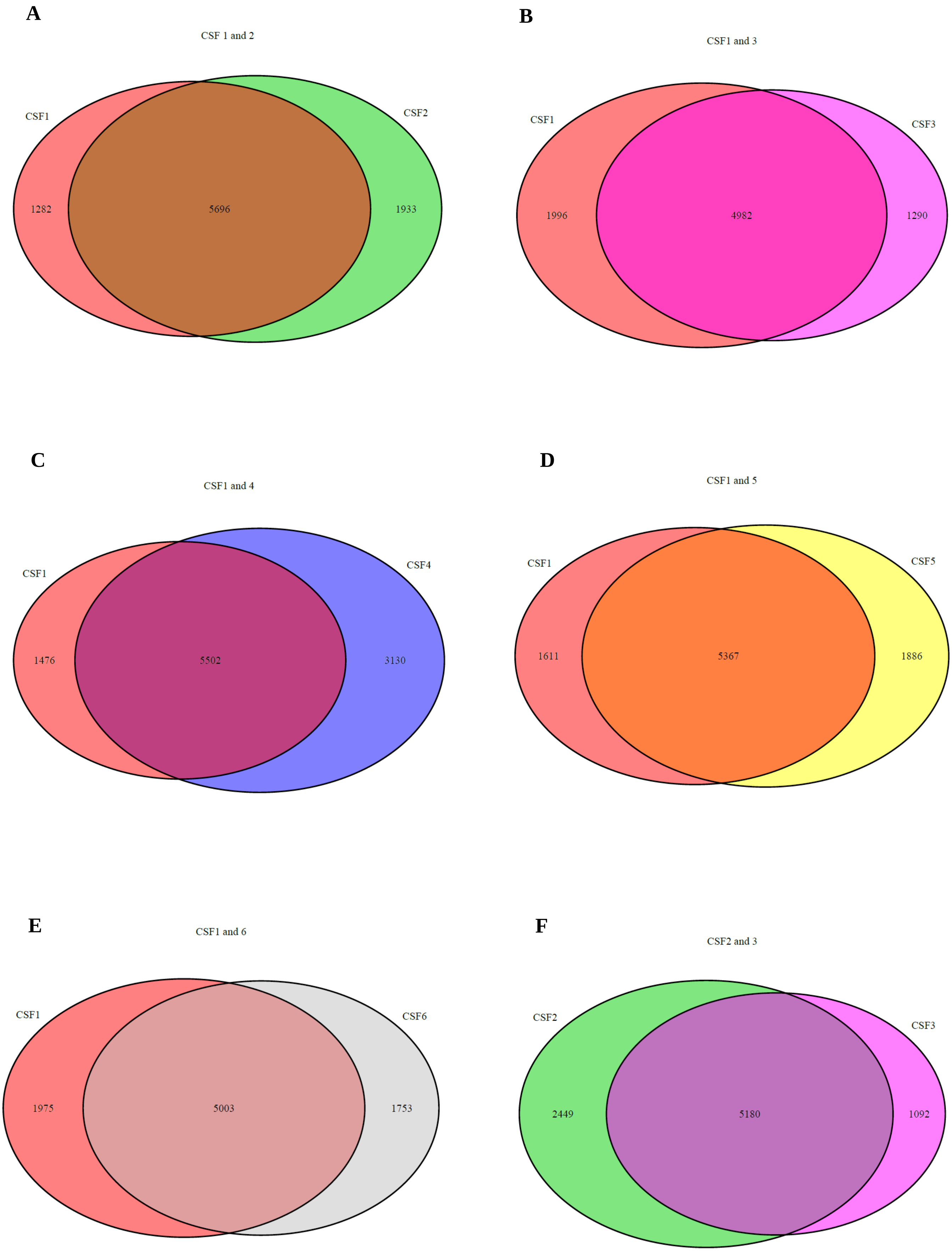

Additional figure 2. Peptides common between two samples. Venn diagrams show common proteins between any two individual CSF samples. The average percentage of common peptides was 74%.

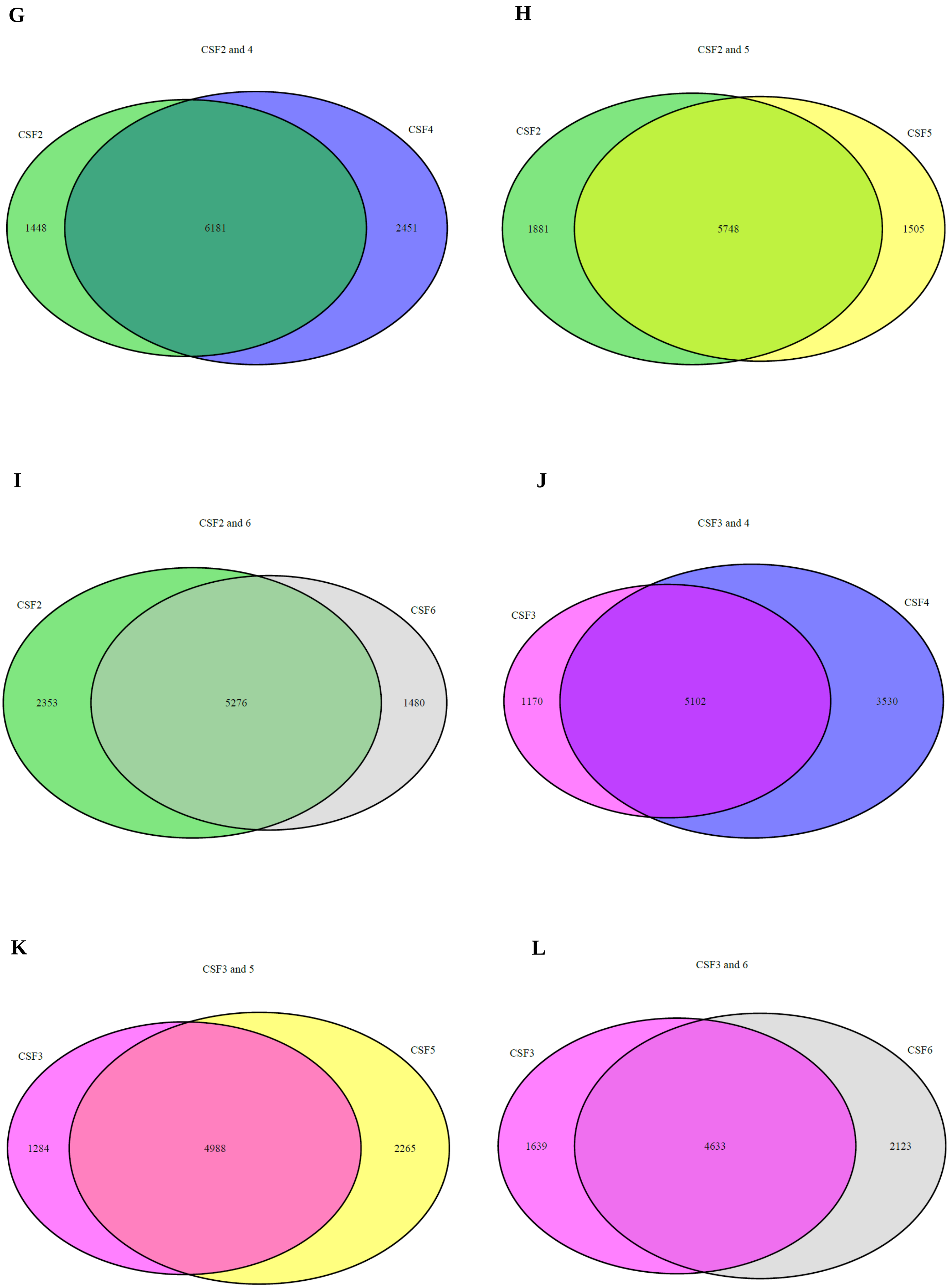

Additional figure 2, continued.

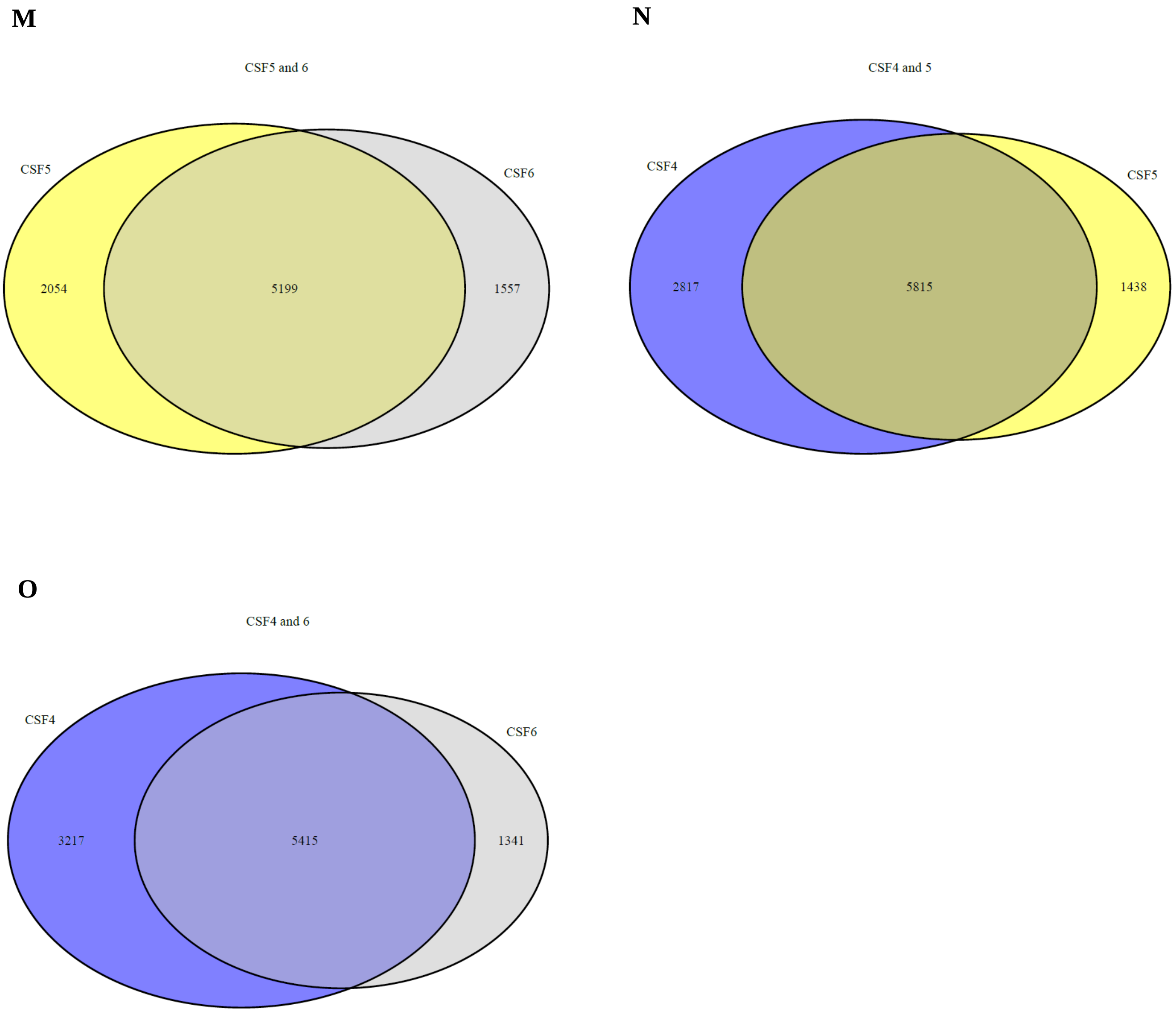

Additional figure 2, continued.
