# Supplementary material for: Identification of brain-enriched proteins in the cerebrospinal fluid proteome by LC-MS/MS profiling and mining of the Human Protein Atlas
Source: Clin Proteomics. 2016 May 15;13:11. doi: 10.1186/s12014-016-9111-3 (PMC4868024; doi:10.1186/s12014-016-9111-3)
Supplement: Supplementary file 6 — 10.1186/s12014-016-9111-3 KLK6 selected reaction monitoring (SRM) assay. [file 12014_2016_9111_MOESM6_ESM.pdf]

Supplementary method

KLK6 selected reaction monitoring (SRM) assay

Post-mortem brain tissue samples were obtained with Research Ethics Board approval from the University Health Network, Toronto, Canada. Frozen tissue sections from several regions (frontal cortex, substantia nigra and cerebellum) from three control patients (diagnosed with non-metastatic colon cancer, cardiovascular disease, or heart failure) were first homogenized in liquid nitrogen, using mortar and pestle, following the homogenization in 50 mM ammonium bicarbonate with Polytron PT3100 homogenizer (Capitol Scientific, USA) at 15,000 rpm, for 30 s and sonicated on ice three times for 10 s with MISONIX immersion tip sonicator (Q SONICA LLC, USA). The samples were centrifuged at 15,000 g at 4 °C for 10 min; supernatants were collected, and samples adjusted to have the equal total protein amount. In addition, CSF pool of non-pathological samples was prepared and subjected to mass spectrometry preparation. For SRM-based quantification 10 µg of total protein was prepared from tissue extracts and CSF pool, denatured with 0.05% RapiGest, reduced using 5 mM dithiothreitol (40 min at 60 °C) and alkylated with 15 mM iodoacetamid (1 hour, 22 °C, in the dark). Heavy labelled synthetic KLK6-proteotypic peptide (Spike Tides TQL, JPT Peptide Technology, Berlin, Germany) was spiked into each sample (10 fmol and 20 fmol/injection for tissue extracts and CSF pool, respectively), after which samples were digested with trypsin, at the enzyme to protein ratio of 1:10 and 1:30 for tissue extracts and CSF pool, respectively at 37 °C overnight. Peptides were then purified by extraction with OMIX C18 tips, separated by liquid chromatography, EASY-nLC system, ionized with nano-electrospray ionization, and analysed using TSQ Vantage and Quantiva mass spectrometer (Thermo Fisher Scientific, USA). Proteotypic peptide LSELIQPLPLER was used for KLK6 quantification. Only three most intense and specific fragment ion transitions (transitions m/z= 965.6, 852.5, 724.4 for light peptide m/z 704.4 and transitions m/z= 975.6, 862.5, 734.4 for heavy peptide m/z 739.9) were used for quantification. The analysis was done using PinPoint (Thermo Fisher Scientific, USA) and Skyline (University of Washington) software.
