# Supplementary material for: Identification of brain-enriched proteins in the cerebrospinal fluid proteome by LC-MS/MS profiling and mining of the Human Protein Atlas
Source: Clin Proteomics. 2016 May 15;13:11. doi: 10.1186/s12014-016-9111-3 (PMC4868024; doi:10.1186/s12014-016-9111-3)
Supplement: Supplementary file 7 — 10.1186/s12014-016-9111-3 KLK6 concentration in brain tissue extracts and CSF pool. Brain tissue extracts and CSF pool were subjected to mass spectrometry sample preparation and analyzed using TSQ Vantage (brain tissues) and TSQ Quantiva (CSF) mass spectrometers. One-way ANOVA and Bonferroni’s Multiple Comparison Test was performed with GradPad Prism between brain regions, n = 3, *p < 0.05. Data are shown as mean ± standard error of the mean (SEM). TP- total protein, SNc- substantia nigra. [file 12014_2016_9111_MOESM7_ESM.pdf]

Additional figure 3.

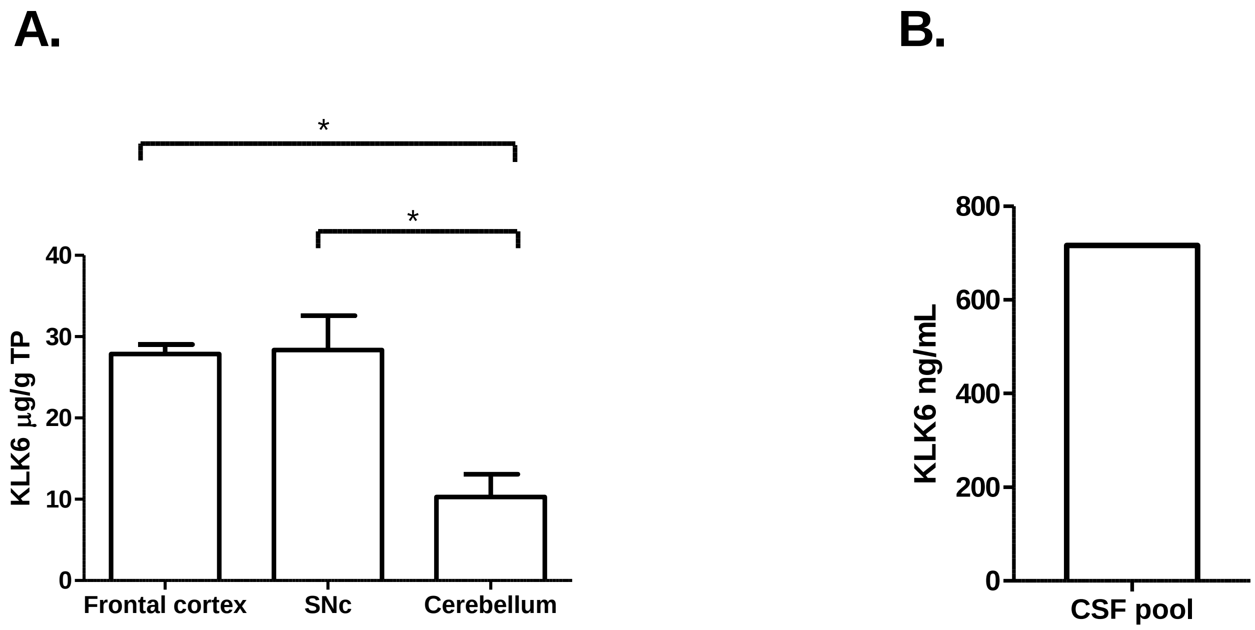

Additional Figure 3. KLK6 concentration in brain tissue extracts and CSF pool. Brain tissue extracts and CSF pool were subjected to mass spectrometry sample preparation and analyzed using TSQ Vantage (brain tissues) and TSQ Quantiva (CSF) mass spectrometers. One-way ANOVA and Bonferroni's Multiple Comparison Test was performed with GradPad Prism between brain regions, n=3, *p<0.05. Data are shown as mean +/- standard error of the mean (SEM). TP- total protein, SNc- substantia nigra.
